# Development of *Tenebrio molitor* (Coleoptera: Tenebrionidae) on Poultry Litter-Based Diets: Effect on Chemical Composition of Larvae

**DOI:** 10.1093/jisesa/ieaa145

**Published:** 2021-01-22

**Authors:** Luciana Barboza Silva, Reneton Gomes de Souza, Sandra Ribeiro da Silva, Alisson da Costa Feitosa, Elainy Cristina Lopes, Stelio Bezerra Pinheiro Lima, Leilane Rocha Barros Dourado, Bruno Ettore Pavan

**Affiliations:** 1 Department of Agricultural Sciences, Federal University of Piauí, Bom Jesus, Piauí, Brazil; 2 Departamento de Fitotecnia, Universidade Estadual Paulista, Ilha Solteira, São Paulo, Brazil

**Keywords:** larval growth, mealworm, protein source, nutrition, organic waste

## Abstract

In order to investigate a low-cost and sustainable food source, the present study evaluated the use of poultry litter for rearing *Tenebrio molitor* Linnaeus, 1758 (Coleoptera:  Tenebrionidae). The experiment was performed with five diets containing increasing levels of poultry litter (0, 25, 50, 75, and 100%) replacing the control diet and five replicates with 50 larvae per sample unit. Larval growth and development were evaluated and the chemical compositions of diet and *T. molitor* larvae were determined. Larval development and reproduction efficiency of *T. molitor* were similar in all treatments. The sole use of poultry litter to feed *T. molitor* reduced the crude protein of flour by only 8%. Including 50% or more poultry litter in the standard diet is the best-suited formulation for larvae production and incorporation of minerals in the larvae. Mealworm can be grown successfully on diets composed by poultry litter, the diet did not affect survival, growth, and development; however, studies spanning several insect generations should be performed to determine the effects of diet composition on adult fecundity. The knowledge acquired using poultry litter to feed *T. molitor* will be useful to carry out new research, in addition to evidencing the possibility of low-cost mass rearing of these larvae.

Due to the projections of accelerated growth of world population, it is essential to increase the production of proteins from animal and plant origin. Insects have been used as an alternative protein source for humans (entomophagy) and other animals because of their greater efficiency in food conversion and less environmental impact (Van Huis et al. 2013, Van Huis 2013, Van Huis 2020). Edible insect larvae contain an average of 54% protein, including essential amino acids and vitamin and mineral properties, which are important characteristics when considering their use as protein source in animal feeding (Sánchez-Muros et al. 2014, Van Huis et al. 2015, Kouřimská and Adámková 2016, Kim et al. 2019).

The yellow mealworm*, Tenebrio molitor* Linnaeus, 1758, is one of the most promising insects as a source of protein for animal feeding at its larvae stage and the chemical composition of its larvae has been extensively researched. This species is one of the largest stored-product beetles (adult body length between 12 and 20 mm), which are commonly found infesting stored agricultural products (Hagstrum et al. 2013). It is a cosmopolitan stored-product pest occurring in a diversity of facilities and commodities, mainly grains and related amylaceous commodities, such as four, bran and pasta (Hagstrum et al. 2013). However, its use in animal feeding is still slightly explored because production at a commercial scale is economically unfeasible due to the high cost of diets required for insect rearing (Nowak et al. 2016, Dreassi et al. 2017, Janssen et al. 2017, Paul et al. 2017). The development of diets capable of supporting and, ideally, maximizing insect growth and development has been identified as one of the major challenges for the insect-producing industry in terms of achieving an efficient, cost-effective, and sustainable insect production for food and feed (Jensen et al. 2017b, Heckmann et al. 2018).

Larvae of *T. molitor* are traditionally fed with bran or wheat flour, oat or corn, and supplemented with protein sources, such as poultry feed and powdered milk. Fruits, vegetables (carrots, potatoes, lettuce, and chayote) can also be included in the diet as a source of moisture and grain residues (Nguyen et al. 2015). This type of diet increases the cost of insect production, making their production and use for animal feeding at a large-scale impossible. However, the larvae can also feed on organic waste, with the potential of recycling lost nutrients by incorporating residual amino acids, fatty acids from manure, and organic waste into their biomass (Oonincx et al. 2015). Further research is needed to satisfy consumers who are concerned with preservation of food safety when using insects reared on different organic materials. These larvae constitute an excellent source of protein and fat, which can be incorporated into monogastric animal feed (Ramos-Elorduy et al. 2002, Veldkamp et al. 2012, Makkar et al. 2014, Henry et al. 2015, Yoo et al. 2019).

Thus, it is essential to carry out studies with alternative sources to create an economically feasible and sustainable diet for *T. molitor*, once the information available to date is not exhaustive; moreover, data on the effect of individual feeding substrates that could serve as *T. molitor* diet components are limited. Poultry litter on the floor of poultry houses is a mixture of excreta, feathers, and feed residues and has high contents of nitrogen, fiber, energy, and minerals (Lana et al. 2010). Adding this residue from poultry production into diet of *T. molitor* larvae will make mass rearing possible, reduce larvae production costs, in addition to constituting an ecological engineering tool to minimize environmental impacts of residues of large agricultural production. The work of the Leek (2016) focused on housefly and trials were running on a poultry farm bioconverting the poultry litter material to insect protein for poultry and aquatic birds. Although the project concept was a success, legislative restrictions on the production and use of insects has meant that outcomes could not yet be commercialized.

Studies of different diets, chemical compositions, and insect development have been accomplished, but results from using poultry litter are scarce (Sheppard et al. 1994, Leek 2016). The objective of this study was to evaluate the growth performance and chemical composition of *T. molitor* larvae fed with diets containing poultry litter.

## Materials and Methods

The experiments were performed at the Plant Protection Laboratory, from the Federal University of Piauí, Campus Professora Cinobelina Elvas - Bom Jesus, PI (09°04″28′S, 44°21″31′W).

### Poultry Litter

The poultry litter was obtained from a commercial production farm of broilers and composed of rice husks and poultry residues from a single cycle. Before using the poultry litter to rear *T. molitor*, it was autoclaved at a pressure of 1.2 atm and 104°C for 60 min, dried in a forced circulation oven at 65°C, and ground with a knife mill.

### Experimental Design

Reared *T. molitor* larvae from the Plant Protection laboratory were used. While performing the experiments, the insects were kept at 28°C, 55% RH, and 12 h photoperiod. A completely randomized design was adopted with five treatments and five replicates, with 50 larvae per experimental unit in plastic containers (30 × 15 × 10 cm).

### Preparation of Diets

The *T. molitor* colony used was established in 2018 from a stock colony provided by the ABRACI (Brazilian Association of Insect Breeders), and since then has been continuously grown in the Laboratory of plant protection of the Federal University of Piaui.

The experimental treatments consisted of a control diet (0%) containing wheat bran (30%), oats (30%), chicken feed (20%) (feed composition for broiler at the final stage of development), powdered milk (10%), and barley (10%), and four test diets with 25, 50, 75, and 100% poultry litter replacing the control diet. Diets were stored at −20°C until the experiments were implemented.

### Larval Development

Fifty newly hatched larvae were transferred to plastic containers (30 × 15 × 10 cm) covered with ‘voil’ fabric for aeration. Each container received 30 g of diet and 10 g of *Sechium edule* (Jacq.) Swartz, 1800, as a source of moisture, replaced every 2 d. Quantitative evaluations of the larvae occurred every 14 d, for a period of 93 d. Adults were transferred to a new container with new diet to evaluate oviposition for 30 d. The evaluated parameters were: larval weight, development period (days), survival (%), pupal number (*N*), total of emerged adults (*N*), efficiency of larva/adult formation, total of F_1_ generation larvae, and the instantaneous population growth rate.

### Analysis of the Chemical Composition of Diet and Larvae (*T. molitor*)

When the experiment was finishing, the larvae were not fed for 48 h, dehydrated in a forced circulation oven at 65°C for 72 h, and ground with a knife mill to produce *T. molitor* flour. A sample of each diet and *T. molitor* flour, from all treatments, were used for the analyses of dry matter (DM), mineral matter (MM), crude protein (CP), organic matter (OM), ether extract (EE), calcium (Ca), magnesium (Mg), phosphorus (P), potassium (K), zinc (Zn), copper (Cu), manganese (Mn), and iron (Fe), which were performed according to AOAC (1990).

### Statistical Analysis

The data were submitted to tests of normality of errors and homogeneity of variances. Subsequently, an analysis of variance was performed using the *f* test (*P* < 0.05) and the treatment averages were compared using the Student Newman Keuls test (SNK) with the software SAS version 9.2 (2008). Additionally, a Pearson correlation and path analysis were performed to assess possible correlations between the chemical composition of the larvae of *T. molitor* reared on a diet supplemented with poultry litter and number F_1_ generation larvae, the magnitudes of the correlation coefficients were classified according to the criteria proposed by Carvalho et al. (2004): null = 0; weak = 0.1 to 0.30; mean = 0.31 to 0.60; strong = 0.61 to 0.90; very strong = 0.91 to 1; these analyses were completed with the genetic statistical software GENES (Cruz 2013, 2016)

The population’s instantaneous growth rate was calculated according to the equation: *ri* = [ln (Nf / N0) / ΔT], where Nf = final number of insects, N0 = initial number of insects, and ΔT = time variation (number of days the test was performed). A positive *ri* value indicates population growth, *ri* = 0 means that the population is stable, and a negative *ri* value indicates population decline until extinction, when Nf = 0 (Walthall and Stark 1997).

## Results

### Evaluation of the Larval Development of *T. molitor*

The mortality of *T. molitor* larvae were approximately 70% in all treatments. The efficiency of *T. molitor* larvae to attain the adult stage was not influenced by the diets tested, since of the number of larvae at the beginning of the experiment and the number of adults at the end was statistically equal (Table 1). The total larval development time (days) was significantly higher in diets with 75 and 100% poultry litter. Progeny production varied significantly among the treatments, with the highest values for the insects fed with 50 and 75% poultry litter, and the instantaneous rate of population growth was statistically equal for all treatments.

**Table 1.** Average development time, survival at larvae, pupal number, adult number, and percentage of successfully emerged adults of *Tenebrio molitor* fed with diets containing different levels of poultry litter

Variable	Poultry litter inclusion level (%)				
	0	25	50	75	100
Larvae weight (g)	4.0a ± 0.2	4.2a ± 0.5	4.3a ± 0.1	3.8a ± 0.7	4.3a ± 0.8
Development time (days)	77.0a ± 4.2	79.0a ± 3.2	82.0b ± 2.3	92.0c ± 3.6	93.0c ± 2.4
Survival larvae (%)	67.6a ± 9.5	81.3a ± 11.5	66.8a ± 7.7	74.9a ± 10.6	72.8a ± 7.6
Pupae (N°)	33.8a ± 3.6	40.6a ± 2.0	33.4a ± 4.3	37.4a ± 2.9	36.4a ± 3.1
Adults (N°)	32.2a ± 3.1	37.8a ± 3.1	32.4a ± 3.0	35.2 ± 2.8	32.8a ± 2.6
Adults from pupae (%)	95.0a ± 0.04	93a ± 0.06	97a ± 0.05	94a ± 0.07	90a ± 0.1
Efficiency Larva/Adult (%)	64.4a ± 6.4	75.6a ± 6.4	64.8a ± 6.1	70.4a ± 5.7	65.6a ± 5.4
Reproduction (%)	33.3abc ± 7.3	28.0c ± 1.7	41.4ab ± 5.8	46.0a ± 12.3	31.5bc ± 2.4
Larvae F1 (N°)	1058.0b ± 179.8	1058.0b ± 126.4	1340.0ab ± 224.5	1598.0a ± 352.5	1030.0b ± 45.5
ri	0.033a ± 0.002	0.033a ± 0.001	0.035a ± 0.002	0.037a ± 0.002	0.033a ± 0.000

Values are given as mean ± SD. Mean followed by the same letters do not differ by the SNK test at 5% probability.

The number of larvae was higher for insects fed with 50 and 75% poultry litter, respectively; however, a quadratic regression was observed (*y* = 885.123811 + 19.92876*x* − 0.16838*x*^2^, *r*^2^ = 0.54) and it was estimated that the point of greatest number of F1 larvae was when 59.2% poultry litter was included in the *T. molitor* diet.

The calculated population’s instantaneous growth rate shows population growth for all analyzed variables (*ri* positive).

### Chemical Composition of Diet and Larvae (*T. molitor*)

The diet containing 100% poultry litter had higher DM content compared to the other diets, and the lowest value was observed with the 0% poultry litter diet. The MM and the analyzed minerals varied in percentage according to the addition of poultry litter to the diets and, simultaneously, there was a reduction in organic matter (OM) and EE (Table 2).

**Table 2.** Chemical composition of diets with increasing levels of poultry litter used to feed *Tenebrio molitor*

Variables	Poultry litter inclusion level (%)					Mean	CV%	VS_TRAT_	VS_ERROR_
	0	25	50	75	100				
	Nutrients (%)								
DM	89.7c	90.2b	90.0b	90.2b	90.7a	90.2	0.2	0.4**	0.0
MM	5.6e	8.6d	10.9c	13.6b	16.0a	10.9	0.6	49.6**	0.0
CP	15.0a	14.9a	14.9a	14.3a	14.9a	14.8	5.6	0.2^ns^	0.6
OM	93.7a	90.5b	87.9c	84.9d	82.4e	87.9	0.1	59.9**	0.0
EE	5.3a	4.9a	3.2b	2.1c	1.0d	3.3	16.0	9.8**	0.2
	Minerals (mg kg^−1^)								
Ca	11.2b	12.1b	24.2a	22.7a	25.6a	19.1	12.5	144.4**	5.7
Mg	4.5e	5.4d	6.6c	7.6b	8.4a	6.5	4.0	7.7**	0.0
P	7.3d	9.6c	11.1b	11.6ab	12.6a	10.4	5.6	12.3**	0.3
K	14.9e	26.3d	55.8c	73.2b	109.2a	55.9	1.9	4273.0**	1.2
Zn	74.8e	133.9d	246.1c	281.0b	405.8 a	208.0	0.1	40377.0**	0.1
Cu	7.9d	21.0c	21.4c	26.4b	36.0a	22.5	4.3	309.5**	0.9
Mn	78.1c	90.1c	146.1ab	136.4b	169.2a	119.0	10.1	3737.0**	144.7
Fe	77.9e	148.0d	204.7c	273.6b	359.1a	213.0	11.8	35676.0**	633.0

CV, coefficient of variation; VS_TRAT_, treatment variation source; VS_ERROR_, error variation source; R, regression; Ca, calcium; Mg, magnesium; P, phosphorus; K, potassium; Zn, zinc; Cu, copper; Mn, manganese; Fe, iron; Ns, *, ** not significant, significant at 5 and 1%, respectively, by the F test; averages followed by the same letters do not differ by the SNK test at 5% probability.

The DM of larvae flour increased with the addition of poultry litter and the CP was lowest when the 100% poultry litter was used. The highest EE was observed when 25% poultry litter was provided, and the lowest was observed with 75% (Table 3).

**Table 3.** Chemical composition of the flour of larvae fed with diets containing increasing levels of poultry litter

Variables	Poultry litter inclusion level (%)					Mean	CV%	VS_TRAT_	VS_ERROR_
	0	25	50	75	100				
	Nutrients (%)								
DM	86.7b	86.7ab	85.9ab	87.6ab	88.4a	87.1	0.8	2.8*	0.5
MM	4.5a	4.0a	4.5a	6.1a	5.0a	4.8	2.0	1.8^ns^	0.9
CP	48.1a	45.8a	46.0a	47.3a	40.2b	45.5	3.2	28.7**	2.1
OM	95.4a	95.9a	95.4a	93.8a	94.9a	95.1	1.0	1.8^ns^	0.9
EE	31.9bc	34.2a	31.3c	27.2d	32.8b	31.5	1.8	21.0**	0.3
	Minerals (mg kg^−1^)								
Ca	0.5e	21.5d	25.3c	28.7b	30.5a	21.3	3.1	440.1**	0.4
Mg	2.0d	6.0c	10.2b	9.4b	12.1a	8.0	7.6	47.5**	0.3
P	8.3c	3.4d	9.2b	9.8b	10.3a	8.2	5.4	25.2**	0.1
K	30.2c	45.8a	29.9c	40.7b	23.8d	34.1	1.6	240.1**	0.2
Zn	100.3e	121.7d	130.3c	136.3b	145.5a	126.8	1.0	884.6**	1.8
Cu	10.5a	11.7a	2.2b	1.7b	1.8b	5.6	7.9	137.2**	0.4
Mn	10.7c	7.0d	15.9b	20.5a	3.3e	11.5	5.3	141.9**	0.3
Fe	56.7b	62.0a	35.0c	5.7d	5.6d	53.2	4.2	4157.6**	5.1

VS, variation source; VS_TRAT_, treatment variation source; VS_ERROR_, error variation source; R, regression; CV, coefficient of variation; Ca calcium; Mg magnesium; P, phosphorus; K, potassium; Zn, zinc; Cu, copper; Mn, manganese; Fe, iron; Ns, *, ** not significant, significant at 5% and 1%, respectively, by the F test; averages followed by the same letters in the columns do not differ by the SNK test at 5% probability.

The chemical composition of larvae flour changed with the addition of poultry litter to the diet, and it is clear that the proportions of 50 and 75% poultry litter kept the composition balanced.

A strong negative correlation was observed between CP and the minerals Mg and Zn, and positive with Mn and Fe (Table 4). The CP also showed a mean positive correlation with Ca and a mean negative correlation with Cu. There was a very strong positive correlation between Zn and Ca and Mg, and a negative one between K and P. Concerning Fe, there was a very strong positive correlation with Cu, strong with K and Mn and a strong negative correlation with P. The very strong negative correlation between OM and MM indicates that these variables are inversely proportional, that is, the increase in OM promotes the reduction of MM. The EE had a very strong negative correlation with Mn, strong with Fe and MM and moderate with Cu. There was a strong positive correlation between EE and OM only. The number of larvae showed a strong positive correlation with Mn and negative with EE, revealing the importance of such mineral for the larvae development.

**Table 4.** Pearson’s correlations of the chemical composition of the larvae of *Tenebrio molitor* reared on a diet supplemented with poultry litter

	Ca	Mg	P	K	Zn	Cu	Mn	Fe	DM	MM	CP	OM	EE
Mg	0.93**												
P	−0.01^ns^	0.32^ns^											
K	0.05^ns^	−0.29^ns^	−0.98**										
Zn	0.96**	0.97**	0.18^ns^	−0.17^ns^									
Cu	−0.18^ns^	−0.43^ns^	−0.78**	0.76**	−0.27^ns^								
Mn	0.07^ns^	0.02^ns^	−0.23^ns^	0.31^ns^	−0.02^ns^	0.51*							
Fe	−0.10^ns^	−0.33^ns^	−0.74**	0.76**	−0.23^ns^	0.95**	0.73**						
Zn	0.97**	0.96**	0.16^ns^	−0.14^ns^	1.00**	−0.25^ns^	−0.01^ns^	−0.20^ns^					
DM	0.37^ns^	0.39^ns^	0.11^ns^	−0.19^ns^	0.47^ns^	0.02^ns^	−0.28^ns^	−0.11^ns^					
MM	0.28^ns^	0.28^ns^	−0.04^ns^	0.01^ns^	0.32^ns^	0.30^ns^	0.35^ns^	0.34^ns^	0.23^ns^				
CP	−0.55*	−0.64*	−0.45^ns^	0.48^ns^	−0.64**	0.59*	0.61*	0.70**	−0.62*	0.11^ns^			
OM	−0.28^ns^	−0.28^ns^	0.04^ns^	−0.01^ns^	−0.32^ns^	−0.30^ns^	−0.35^ns^	−0.34^ns^	−0.23^ns^	−1.00**	−0.11^ns^		
EE	−0.22^ns^	−0.20^ns^	0.09^ns^	−0.11^ns^	−0.23^ns^	−0.51*	−0.85**	−0.66**	−0.15^ns^	−0.63*	−0.37^ns^	0.63*	
Nº Larvae	0.40	0.34^ns^	−0.12^ns^	0.15^ns^	0.31^ns^	0.30^ns^	0.75**RE	0.47^ns^	0.08^ns^	0.47^ns^	0.16^ns^	−0.47^ns^	−0.76**

Ca, calcium; Mg, magnesium; P, phosphorus; K, potassium; Zn, zinc; Cu, copper; Mn, manganese; Fe, iron; Ns, *, ** not significant, significant at 1 and 5% probability, respectively, by the *t* test.

According to the results from the path analysis, among the characteristics analyzed, the greatest direct effect on the number of larvae is associated with DM in diets (negative association) and MM in larvae flour (positive association) (Table 5). DM has a highly direct negative influence on the number of larvae when the value is greater than two. Therefore, diets with high DM are not recommended to obtain a larger number of larvae. The characters that have a direct positive influence with larvae are CP and MM with values greater than 1. Both characteristics have an indirect association via negative DM, as well as with each other, canceling their direct correlations, which results in low total correlation and could influence the CP to remain negative. However, in direct associations, diets with high levels of CP and MM provided greater numbers of larvae.

**Table 5.** Path analysis for the main larvae number variable of *Tenebrio molitor* and secondary variables of the chemical composition of the larvae flour and the diet DM, MM, CP, OM, and EE

Variable	Diet		Larvae	
	Association	Yield	Association	Yield
DM	Direct	−2.25	Direct	0.19
	Indirect via MM	0.20	Indirect via MM	0.24
	Indirect via CP	1.31	Indirect via CP	−0.04
	Indirect via OM	−0.01	Indirect via OM	−0.08
	Indirect via EE	0.08	Indirect via EE	−0.11
	Total	−0.66	Total	0.19
MM	Direct	1.29	Direct	0.99
	Indirect via DM	−0.35	Indirect via DM	0.04
	Indirect via CP	−0.53	Indirect via CP	−0.09
	Indirect via OM	−0.10	Indirect via OM	−0.36
	Indirect via EE	−0.20	Indirect via EE	−0.23
	Total	0.10	Total	0.36
CP	Direct	1.64	Direct	−0.14
	Indirect via DM	−1.79	Indirect via DM	0.05
	Indirect via MM	−0.42	Indirect via MM	0.67
	Indirect via OM	0.03	Indirect via OM	−0.24
	Indirect via EE	0.19	Indirect via EE	−0.24
	Total	−0.35	Total	0.12
OM	Direct	0.10	Direct	0.36
	Indirect via DM	0.35	Indirect via MS	−0.04
	Indirect via MM	−1.29	Indirect via MM	−0.99
	Indirect via CP	0.53	Indirect via CP	0.09
	Indirect via EE	0.20	Indirect via EE	0.23
	Total	−0.10	Total	−0.36
EE	Direct	0.24	Direct	0.26
	Indirect via DM	−0.78	Indirect via DM	−0.08
	Indirect via MM	−1.07	Indirect via MM	−0.87
	Indirect via CP	1.30	Indirect via CP	0.13
	Indirect via OM	0.08	Indirect via OM	0.32
	Total	−0.23	Total	−0.24

Regarding the larvae flour, the only characteristic that stood out as having a direct effect was MM. The other characteristics have total correlations and a direct effect of low magnitude, which is often the greatest indirect association route by MM. MM directly influenced the number of larvae and indirectly influenced the other characteristics analyzed. Path analysis is important because it breaks down the correlation analysis in direct and indirect effects, which provides greater security when choosing the characteristics that should be better worked out if the final goal is reached, in this case, the largest production of larvae.

## Discussion

This study compares the effects of organic waste, poultry litter, on *T. molitor.* Effects on survival and development time, efficiency larva/adult, larvae F1 (progeny), as well as insect and diet chemical composition, are reported. Whereas all diets were accepted by the *T. molitor*, development times were not affected by poultry litter, the same was true for survival rate. Diet plays an important role for insect growth and performance; therefore, the development of an effective artificial diet has been identified as a crucial component of insect-producing systems, such as the mass production of beneficial arthropods in general (Chen et al. 2014, Morales-Ramos et al. 2014, Huynh et al. 2019, Rumbos et al. 2020). In the case of *T. molitor*, considerable effort has been focused on the development of artificial diets that would maximize the biomass production of highly nutritious larvae, with a concomitant reduction in their developmental time as well as low-cost diet.

According to the data obtained, all treatments used to feed *T. molitor* resulted in larval development similar to the control diet. Some insects have the ability to regulate nutrient intake through self-selection of food (Morales-Ramos et al. 2010, 2011, 2020). This type of selection varies based on the nutritional requirement of each species and maintains the balance of digestible carbohydrates and proteins (Behmer 2009, Morales-Ramos et al. 2011), lipids and vitamins (Schiff et al. 1988). Self-selection can also occur according to the stage of the insect (Deans et al. 2017, Fowles and Nansen 2019). In this study, we observed that *T. molitor* larvae sought to consume the ideal proportion of the mixture of the control diet with the poultry litter for their nutritional balance.

Different ingredients can be used to compose the diet of *T. molitor*, since the diets that contain a mixture of various ingredients could potentially be relatively more nutritionally balanced to attend the nutritional requirements of insects (Rumbos et al. 2020). Thus, mixtures of poultry litter with the control diet in different proportions allow us to determine the best combinations of these components (Sheppard et al. 1994, Leek 2016). Our results build on previous studies showing that feed composition has a major impact on *T. molitor* growth performance. In our study, the highest efficiency of total progeny was produced in diets with 50 and 75% de poultry litter. The amount of food offered to insects may have contributed to the acquisition of the nutrients necessary for their development, the poultry litter being a low-cost resource, allows a large-scale use to feed *T. molitor*. The next steps studying this matter are to evaluate the sanitary issue of using *T. molitor* reared with poultry litter and studies focusing on finding optimized combinations of poultry litter and diet composition, in order to efficiently produce insects that meet the nutritional requirements of animals.

We know that insects are great recyclers of byproducts, Fowles and Nansen (2019) showed a review, about research on the growth performance and feeding conversion of these species, suggesting they alone are not sufficient to fully capitalize on the high diversity of unique organic wastes available for bioconversion.

The chemical composition of the diet with the added poultry litter had higher Ca, P, K, Zn, Cu, Mn, and Fe values. The development of *T. molitor* in the poultry litter was similar to the control diet that is considered efficient for rearing this insect, according to larva-adult viability, number of F_1_ generation larvae, and the instantaneous population growth rate. According to Grangeteau et al. (2018) and Weldon et al. (2019), the quality of the offered diet in the larval phases influences the reproductive performance of adults. This can be evidenced by the path analysis, where DM had a direct effect on the number of adults since the diet with the poultry litter had a higher proportion of solid constituents.

These results are satisfactory, considering that one of the obstacles when using insect flour for human consumption and animal husbandry is the retail price of insects, which is still high compared to animal protein (AHDB 2014). The cost is still high because adequate and low-cost techniques for the development of insects are lacking, and larval growth is influenced by several factors, such as larval stage, food nutrition, and humidity (Morales-Ramos and Rojas 2015, Van Broekhoven et al. 2015), whereas the number of instars (time) of *T. molitor* increases in response to adverse conditions (Esperk et al. 2007). It was verified that larvae reached adulthood and that oviposition of adults was similar between the standard diet and 100% poultry litter.

Insects can supply protein cheaper and more sustainably than conventional livestock. The high efficiency of feed conversion, the need for less water, and their less disease-prone makes insects an adequate source of protein. However, legislation is still an obstacle; although, many countries have started drafting legislation to identify insect species as promising candidates for large-scale breeding and to be used for human consumption and animal breeding, e.g., the European Union (EU) places insects in a new food category (EC 258/1997). The solely use of poultry litter to feed *T. molitor* reduced the CP in the larvae flour by only 8%, although, the values obtained for all diets with poultry litter are within the percentages of CP observed in other studies that evaluated insect flour (40 to 60%) (Yi et al. 2013, Ghosh et al. 2017, Kim et al. 2019). Insect proteins are similar to animal protein and the quality of the protein depends on the composition of amino acids (Kouřimská and Adámková 2016, Inje et al. 2018).

The nutrient with the highest participation in the DM of the larvae flour was CP, followed by EE. According to Ghosh et al. (2017), *T. molitor* is 34% fat, which is similar to the percentage of fat obtained for the larvae reared with 100% poultry litter (32%). Assessing the quality of fat is complex. Saturated fatty acids are a food factor and have their greatest negative effect on LDL cholesterol (low-density lipoproteins). Polyunsaturated fatty acids in flour have many beneficial effects, reduce the risk of coronary heart disease, modulate the metabolism of VLDL (very-low-density lipoproteins) and reduce plasma triglycerides. Compared to conventional foods, fats from insects are clearly superior (Van Broekhoven et al. 2015, Dreassi et al. 2017). In the present study, we observed that the incorporation of EE by *T. molitor* is little influenced by dietary fat, since the EE values of the larvae were similar to the larvae that received 0 and 100% poultry litter.

As for the chemical composition of the flour of larvae fed with poultry litter, values for some minerals were higher than the control diet, which is similar to previous studies that used other diets (Ghosh et al. 2017). Calcium was higher in insects raised with 100% poultry litter. From a nutritional point of view, calcium is of interest in medical science because of its role in osteoporosis and several other chronic diseases, including hypertension and cervical cancer. Calcium is an essential mineral because of its phosphate salts that play essential roles in neuromuscular function and many enzyme-mediated processes, such as blood clotting and bone and tooth formation (Ghosh et al. 2017). The rate of K in *T. molitor* in the treatments was of 25 mg, which makes these larvae potentially valuable compared to conventional animal meat, such as pork (6.0 mg), beef (4.5 mg), and chicken (2.7 mg) (Ghosh et al. 2017, Sogari et al. 2019). Low levels of potassium in humans have been associated with a variety of physiological factors (e.g., respiratory, kidney, hypertension). Unlike phosphorus in plants, in insects, this mineral is readily available, and the amount of P obtained from the larvae in treatments with the poultry litter was higher than in the control diet. The increase in the levels of Ca and P improves the nutritional composition of the meal of larvae of *T. molitor* with the use of the highest levels of poultry litter, this enriches this flour and when used in animal feed improve the supply of these minerals. Copper plays an important role in cellular respiration, biosynthesis of neurotransmitters and the formation of pigments, but it is toxic in excess. The availability of mineral content in *T. molitor* reared with poultry litter is significant, making this insect an alternative to mitigate the risk of malnutrition due to micronutrient deficiency (Ghosh et al. 2017).

The path analysis shows that diets with a high value of CP and MM are the most suitable to obtain a higher number of larvae, and diets with a high content of DM are inefficient for this purpose. DM negatively influences OM and EE, masking its effects mainly for CP. Therefore, efficient diets are those that have a high nutritional value and little organic carbon, containing primarily many minerals and OM with a low carbon/nitrogen ratio, which allows to obtain a greater amount of proteins in relation to organic carbon.

The knowledge acquired from using poultry litter to feed *T. molitor* will be useful for carrying out new research and emphasizes the possibility of mass rearing at a low cost. Including insect meal in the animal meat product industry could alleviate excessive pressure on the commercial breeding of cattle, swine and poultry, and could be an alternative that lowers the environmental impact of producing animal protein. The chemical composition of the poultry litter diet is viable for *T. molitor* mass breeding. Including 50% or more poultry litter in the standard diet provides the best-suited formulation to produce larvae and incorporate minerals in the larvae.
